# Evaluating the responses of forest ecosystems to climate change and CO_2_ using dynamic global vegetation models

**DOI:** 10.1002/ece3.2735

**Published:** 2017-01-17

**Authors:** Xiang Song, Xiaodong Zeng

**Affiliations:** ^1^ International Center for Climate and Environment SciencesInstitute of Atmospheric PhysicsChinese Academy of SciencesBeijingChina; ^2^ Collaborative Innovation Center on Forecast and Evaluation of Meteorological DisastersNanjing University of Information Science & TechnologyNanjingChina; ^3^ University of Chinese Academy of SciencesBeijingChina

**Keywords:** climate change, CO_2_ concentration, dynamic global vegetation model, forest ecosystem, tree fractional coverage

## Abstract

The climate has important influences on the distribution and structure of forest ecosystems, which may lead to vital feedback to climate change. However, much of the existing work focuses on the changes in carbon fluxes or water cycles due to climate change and/or atmospheric CO_2_, and few studies have considered how and to what extent climate change and CO_2_ influence the ecosystem structure (e.g., fractional coverage change) and the changes in the responses of ecosystems with different characteristics. In this work, two dynamic global vegetation models (DGVMs): IAP‐DGVM coupled with CLM3 and CLM4‐CNDV, were used to investigate the response of the forest ecosystem structure to changes in climate (temperature and precipitation) and CO_2_ concentration. In the temperature sensitivity tests, warming reduced the global area‐averaged ecosystem gross primary production in the two models, which decreased global forest area. Furthermore, the changes in tree fractional coverage (Δ*F*_tree_; %) from the two models were sensitive to the regional temperature and ecosystem structure, i.e., the mean annual temperature (MAT; °C) largely determined whether Δ*F*_tree_ was positive or negative, while the tree fractional coverage (*F*_tree_; %) played a decisive role in the amplitude of Δ*F*_tree_ around the globe, and the dependence was more remarkable in IAP‐DGVM. In cases with precipitation change, *F*_tree_ had a uniformly positive relationship with precipitation, especially in the transition zones of forests (30% < *F*_tree_ < 60%) for IAP‐DGVM and in semiarid and arid regions for CLM4‐CNDV. Moreover, Δ*F*_tree_ had a stronger dependence on *F*_tree_ than on the mean annual precipitation (MAP; mm/year). It was also demonstrated that both models captured the fertilization effects of the CO_2_ concentration.

## 1 Introduction

The distribution and features of forest ecosystems are largely determined by climate. On the individual level, climate directly influences seed reproduction and seedling establishment (Renard, Mclntire, & Fajardo, 2016), growth (Carrer & Urbinati, 2004, 2006), leaf area and form (Fisher et al., 2007; Yang et al., 2015), phenology (Pettorelli et al., 2005; Zhang et al., 2003), and longevity of individual plants (Goulden et al., 1998). On the ecosystem level, climate change can alter productivity (Meir, Metcalfe, Costa, & Fisher, 2008; Schwalm et al., 2010; Wang et al., 2016), species composition (Renwick & Rocca, 2015), and regional diversity (Beaumont et al., 2011; Garcia, Cabeza, Rahbek, & Araújo, 2014; Ohlemüller et al., 2008) and can even result in shifts from one ecological state to another (Bush, Hanselman, & Gosling, 2010). Such changes may lead to vital feedback in the water and carbon cycles (Gonzalez‐Meler, Rucks, & Aubanell, 2014); therefore, it is important to explore how climate change influences the structure and functions of forest ecosystems.

Most projections of future climate change refer to temperature and precipitation changes, as well as increasing concentrations of greenhouse gases in the atmosphere. Temperature is the main influencing factor of many ecosystem processes (Badeck et al., 2004) and the carbon balance. For example, Rustad et al. (2001) used meta‐analysis to find that experimental warming of soil temperature in the range 0.3–6.0°C significantly increased soil respiration rate by 20%, net N mineralization rate by 46%, and plant productivity by 19%. Lin, Zhu, Wang, Gong, and Zou (2016) analyzed gross primary production (GPP) and net primary production products during 2000–2010 and leaf area index (LAI; m^2^/m^2^) products during 1981–2011 and found that the air temperature had a significant positive correlation with LAI (*R* ^2^ = .311) and GPP (*R* ^2^ = .189). Meanwhile, it has been discovered that the responses of ecosystems to temperature change are spatially heterogeneous and partly uncertain (Mekonnen, Grant, & Schwalm, 2016; Williams et al., 2010; Willis, Bennett, Burrough, Macias‐Fauria, & Tovar, 2012). Plenty of work has shown that because of temperature limitations, warming favors boreal forests in the form of increases in vegetation cover (Berner, Beck, Bunn, & Goetz, 2013) and northward movement of tree lines. However, for some tropical forests, temperature has a strong negative effect on stem growth by increasing respiration and decreasing photosynthesis due to reduced stomatal conductance (Schippers, Sterck, Vlam, & Zuidema, 2015). Willis et al. (2012) concluded that when regional conditions become warmer and wetter, the biomass and range distribution of trees are likely to increase, while if a transition to warmer and drier conditions occurs, grass or savanna replaces woody vegetation in many regions.

Precipitation is another vital factor, influencing tree growth (Subedi & Sharma, 2013; Voelker, Meinzer, Lachenbruch, Brooks, & Guyette, 2014) and affecting forest population dynamics (Booth et al., 2012; De Steven, 1991). More precipitation during the wettest quarter increases tree diameter growth (Subedi & Sharma, 2013), whereas reductions in photosynthesis occur during droughts, which decrease GPP (Schwalm et al., 2010; Van der Molen et al., 2011). Wu, Dijkstra, Koch, Peñuelas, and Hungate (2011) demonstrated that decreased precipitation suppressed aboveground biomass, whereas increased precipitation stimulated aboveground and belowground biomass. Moreover, the CO_2_ concentration is the third factor related to climate change because it is expected to have a direct fertilization effect (Norby & Zak, 2011) and lead to warming. Kimball (1983) had estimated that a doubling of the CO_2_ concentration, all else constant, will increase growth and yield approximately 34 ± 6% in C3 plants and 14 ± 11% in C4 plants. However, elevated CO_2_ does not always have a positive relationship with biomass and growth, and its fertilization effects partly depend on forest age (Körner et al., 2005) and individual tree size (Kim, Oren, & Qian, 2016).

In recent two decades, dynamic global vegetation models (DGVMs) have become important tools to investigate and predict the rate and direction of changes in global vegetation biomes in response to climate change and rising atmospheric CO_2_ (Cramer et al., 2001; Notaro, 2008; Shafer, Bartlein, Gray, & Pelltier, 2015; Woodward & Lomas, 2004). Some are coupled with climate models to predict climate–vegetation interactions (Sitch et al., 2003; Notaro, Chen, & Liu, 2011), while others are run offline with different scenarios to explore the effects of changes in climate or CO_2_ on vegetation (Ni, Harrison, Prentice, Kutzbach, & Sitch, 2006; Peng et al., 2009; Plattner et al., 2008; Ruosch et al., 2016; Shafer et al., 2015; Sitch et al., 2008; Woodward & Lomas, 2004; Zhang et al., 2015). For example, Cramer et al. (2001) used six DGVMs to investigate the responses of ecosystem carbon to changes in climate and CO_2_ concentration. Woodward and Lomas (2004) used SDGVM (the Sheffield DGVM) to find that a scenario of future global warming resulted in a gradual decline in the terrestrial carbon sink. Galbraith et al. (2010) used three DGVMs to explore the mechanisms of Amazonian forest biomass changes under climate change; and it was found that high temperature directly increased plant respiration and declined photosynthesis and then led to reduction in forest biomass losses (Galbraith et al., 2010). Furthermore, large uncertainties may exist among different DGVMs. Sitch et al. (2008) used five DGVMs to explore that significant discrepancies were associated with the response of tropical vegetation to drought and boreal ecosystems to elevated temperatures and changing soil moisture status.

Attention has been given to the relationship between terrestrial ecosystems and climate change and atmospheric CO_2_. However, much of the research has focused on the influences of climate change and/or atmospheric CO_2_ on carbon fluxes or water cycles, and few work considered how climate change and CO_2_ influence the ecosystem structure (e.g., fractional coverage change) and which ecosystem types are susceptible to varying climate and CO_2_. Such issues are very important because they have a direct impact on global biogeography, carbon and water cycles, vegetation succession, and the time scale of vegetation ecosystem recovery.

In this work, two DGVMs (a revised version of IAP‐DGVM1.0 and CLM4‐CNDV) were used to investigate the responses of forest ecosystems to climate change with respect to changes in temperature, precipitation, and CO_2_ concentration. The following questions are addressed: (1) Which regions are sensitive to climate change? (2) When the temperature, precipitation, and CO_2_ concentration vary, how do the forest area and fractional coverage change? (3) Which factor has larger influences on the change in *F*_tree_ (Δ*F*_tree_; %), climate or forest ecosystem structure, and how? (4) Which climate conditions favor forest ecosystems in different regions?

## 2 Model Description

### 2.1 A revised IAP‐DGVM1.0

IAP‐DGVM1.0 (Zeng, Li, & Song, 2014) was developed by the Institute of Atmospheric Physics, the Chinese Academy of Sciences, to investigate ecological processes and to study land–atmospheric interactions. It involves photosynthesis, respiration, phenology, individual carbon allocation, competition, survival and establishment, mortality, litter decomposition, soil respiration, and fire disturbance. IAP‐DGVM1.0 has been coupled with CLM (Oleson et al., 2004; Zeng et al., 2014) and the Common Land Model (Dai, Dickinson, & Wang, 2004; Dai et al., 2003; Zhu, Zeng, Li, & Song, 2014) to describe the major regions of tree, shrub, grass, and bare soil under current climatic conditions (Zeng et al., 2014; Zhu et al., 2014), as well as vegetation–climate relationships.

Subsequently, a revised IAP‐DGVM1.0 introduced the effects of soil moisture during the growing season on the establishment rate of woody plant functional types (PFTs) in the establishment scheme (Song, Zeng, Zhu, & Shao, 2016). When coupled with CLM3, compared with the default IAP‐DGVM1.0, the revised version reduced biases in forest fractional coverage in approximately 78.8% of the global grid cells, especially in arid and semiarid regions and the transition zones of forests (Song et al., 2016). In this work, the revised IAP‐DGVM1.0 coupled with CLM3 is used and abbreviated as IAP‐DGVM in the following sections.

### 2.2 CLM4‐CNDV

The Community Land Model 4.0 (CLM4; Oleson et al., 2010) builds on CLM3.5 with the introduction of a carbon and nitrogen cycle model. CLM4 includes an option to run CLM4CN as a DGVM (CLM4‐CNDV), and the modules of DGVM follow the prior versions of CLM‐DGVM without major modifications. CNDV changes the CN framework only as needed to simulate biogeography updates, including light competition, establishment and survival, as well as mortality. All other ecosystem processes (such as individual allocation, phenology, and fire) are handled by CN (Castillo, Levis, & Thornton, 2012; Oleson et al., 2010).

## 3 Experimental Design

Two types of global offline simulations were conducted: one using IAP‐DGVM coupled with CLM3 and the other using CLM4‐CNDV. All simulations were forced circularly with 50 years of reanalysis surface atmospheric fields (1950–1999) from Qian, Dai, Trenberth, and Oleson (2006). IAP‐DGVM ran for 800 years with T62 resolution (79 × 192 grid cells covering 60°S–90°N) to equilibrium and then restarted for another 50 years with the default atmospheric fields (control case) and climate change (i.e., with changes in temperature, precipitation, or CO_2_ concentration) in several separate cases: (1) temperature ±1°C, ±2°C, and ±3°C at each time step (abbreviated as mean annual temperature [MAT] ± 1°C, MAT ± 2°C, and MAT ± 3°C, respectively); (2) precipitation increased or decreased by 15% (abbreviated as MAP115 and MAP085); and (3) doubling the CO_2_ concentration (2CO_2_). For CLM4‐CNDV, the 20th‐century control simulation documented by Bonan and Levis (2010) (initial conditions supplied with the CCSM4 release) was used as the initial data to run CLM4‐CNDV for 600 years to equilibrium with 96 × 144 grid cells. The simulation was then restarted for the same ten cases with climate change and one control case, as IAP‐DGVM. The last 50 years of simulation results were analyzed. In each simulation, only one climate factor was changed, and the others remained at the default settings. For simplicity, variables from the control cases of the two models were marked “ctrl” in the subscript.

In IAP‐DGVM and CLM4‐CNDV, natural plants are classified into 12 PFTs according to their physical, phylogenetic, and phenological characteristics, including seven trees (Table 1), two shrubs, and three grasses, in which trees have the highest hierarchy for the competition of establishment. Therefore, the simulation performance of tree PFTs has a direct influence on other PFT simulations, and this work mainly focused on how climate change influences forest coverage and its relevant variables using IAP‐DGVM and CLM4‐CNDV. The definition of fractional coverage and related parameterizations is shown in Appendice S1.

**Table 1:** The list of seven tree plant functional types in IAP‐DGVM and CLM4‐CNDV

Full Name	Abbreviation
Trees	
Needleleaf evergreen temperate	NEM‐Tr
Needleleaf evergreen boreal	NEB‐Tr
Broadleaf evergreen tropical	BET‐Tr
Broadleaf evergreen temperate	BEM‐Tr
Broadleaf deciduous tropical	BDT‐Tr
Broadleaf deciduous temperate	BDM‐Tr
Broadleaf deciduous boreal	BDB‐Tr

## 4 Results

### 4.1 The effects of temperature change on forest ecosystems

#### 4.1.1 Comparison among different sensitivity tests of temperature change

##### Global distribution of regions sensitive to temperature change

**Figure 1:**
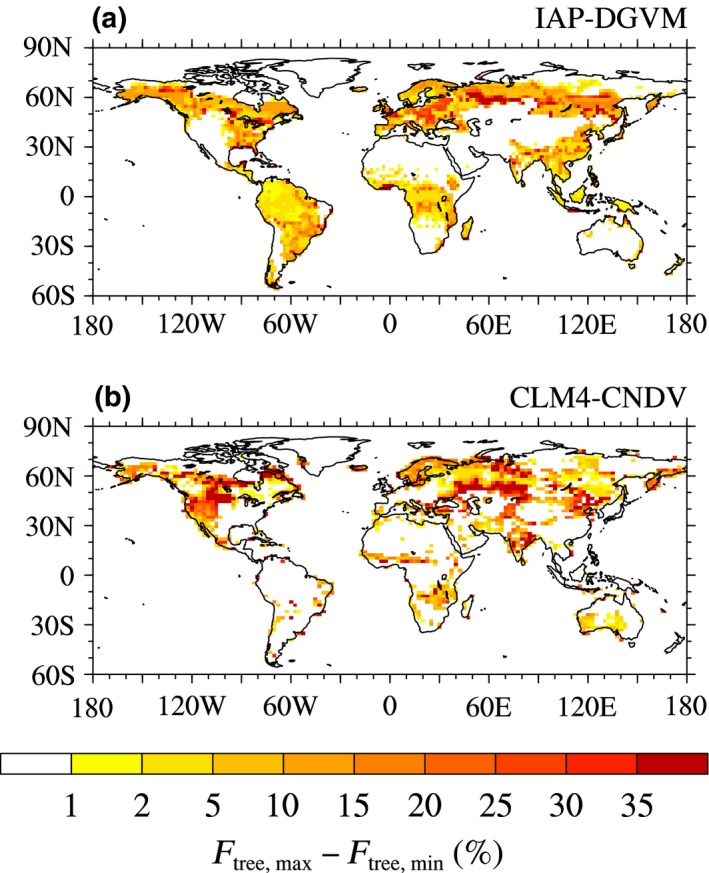
The global distribution of differences in the maximum tree fractional coverage (*F*_tree,max_; %) and the minimum tree fractional coverage (*F*_tree,min_; %) among seven temperature sensitivity tests for (a) IAP‐DGVM and (b) CLM4‐CNDV

First, to investigate which areas are sensitive to temperature change, the global distribution of differences between the maximum tree fractional coverage (*F*_tree,max_; %) and the minimum tree fractional coverage (*F*_tree,min_; %) from seven temperature sensitivity tests is shown in Figure 1. In IAP‐DGVM, most forest regions were influenced by temperature change, and the most sensitive areas were distributed in the core areas of forests, especially in boreal forests, where the amplitude of the *F*_tree_ change was approximately 10%–20%, exceeding 35% in some grid cells. In CLM4‐CNDV, boreal regions also had significant sensitivity to temperature change; however, the most influenced areas were distributed in the transitional areas of boreal forests, the peripheral zones of tropical forests, and some semiarid or arid regions (e.g., western America).

##### The influence of temperature change on gross primary production

**Figure 2:**
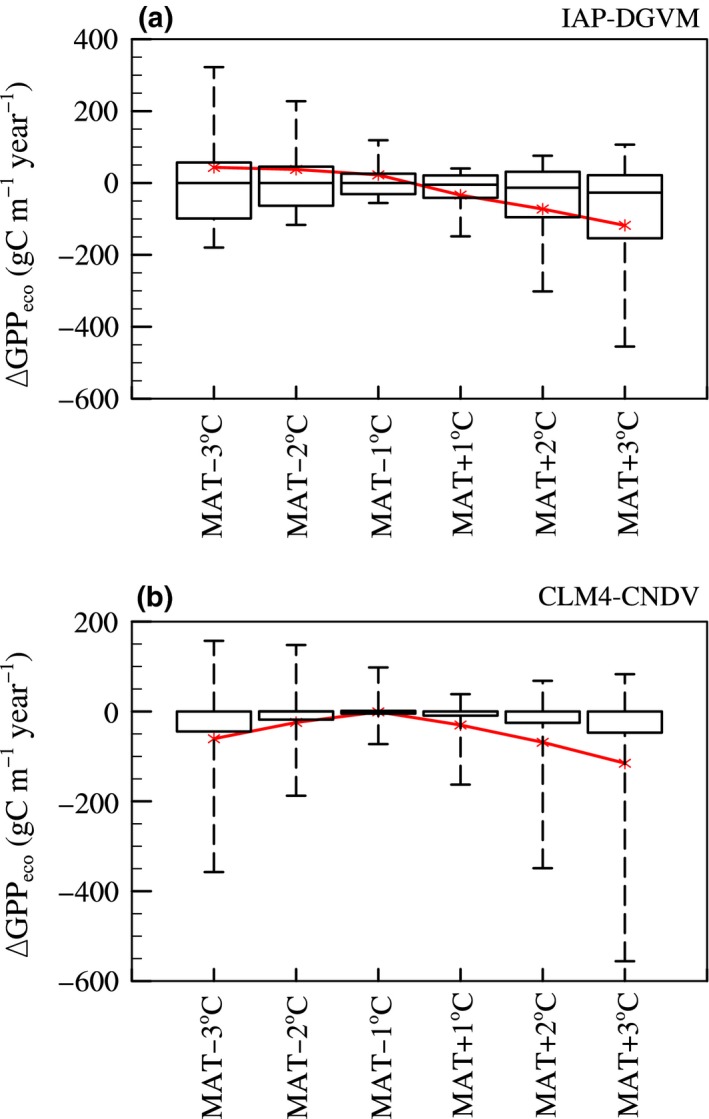
Comparison of the change in ecosystem gross primary production (ΔGPP_eco_; gC m^−1^ year ^−1^) among six temperature change sensitivity tests by IAP‐DGVM and CLM4‐CNDV, respectively. The boxplots denote the 10th, 25th, median, 75th, and 90th percentiles, and the red star line is the global area‐averaged ΔGPP_eco_

Temperature change influences terrestrial ecosystems in various ways, and one of the most direct ways is affecting the GPP of the ecosystem (GPP_eco_; gC m^−2^ year^−1^; see Appendice S1), leading to changes in ecosystem characteristics and structure. Therefore, the changes in forest ecosystem GPP (ΔGPP_eco_; gC m^−1^ year^−1^, i.e., GPP_eco_ in cases of temperature change—GPP_eco_ in case of control simulation for each model) with different cases were investigated, where the boxplots showed the 10th, 25th, median, 75th, and 90th percentiles, and the red star line was the global area‐averaged value ($\bar{\Delta {\text{GPP}}_{\mathrm{eco}}}$; gC m^−2−^year^−1^; Figure 2; it should be declared that the median lines in Figure 2b were almost near the zero line, so they could not be seen clearly). Overall, the greater the MAT change was, the larger the change in GPP_eco_ was, and ΔGPP_eco_ from CLM4‐CNDV usually had a larger standard deviation than that from IAP‐DGVM. For IAP‐DGVM, as MAT changed by −3°C to 3°C, $\bar{\Delta {\text{GPP}}_{\mathrm{eco}}}$ decreased from 43.3 to −117.8 gC m^−2^ year^−1^, while for CLM4‐CNDV, it first increased and then decreased, and the peak appeared at MAT − 1°C (^−^1.0 gC m^−2^ year^−1^). For both models, warming resulted in drier soil moisture and then led to a drop in average GPP_eco_ ($\bar{{\text{GPP}}_{\mathrm{eco}}}$; gC m^−1^ year^−1^). However, the $\bar{{\text{GPP}}_{\mathrm{eco}}}$ simulated by the two models had distinct responses to cooling, i.e., $\bar{\Delta {\text{GPP}}_{\mathrm{eco}}}$from IAP‐DGVM increased when MAT decreased by from −1°C to −3°C ($\bar{\Delta {\text{GPP}}_{\mathrm{eco}}}$increased from 22.5 to 43.3 gC m^−2^ year ^−1^), while $\bar{\Delta {\text{GPP}}_{\mathrm{eco}}}$ from CLM4‐CNDV decreased as MAT decreased ($\bar{\Delta {\text{GPP}}_{\mathrm{eco}}}$fell from −1.0 gC m^−2^ year ^−1^ in the case of MAT − 1°C to −60.7 gC m^−2^ year ^−1^ in the case of MAT − 3°C).

##### Changes in global areas of different forest types

**Figure 3:**
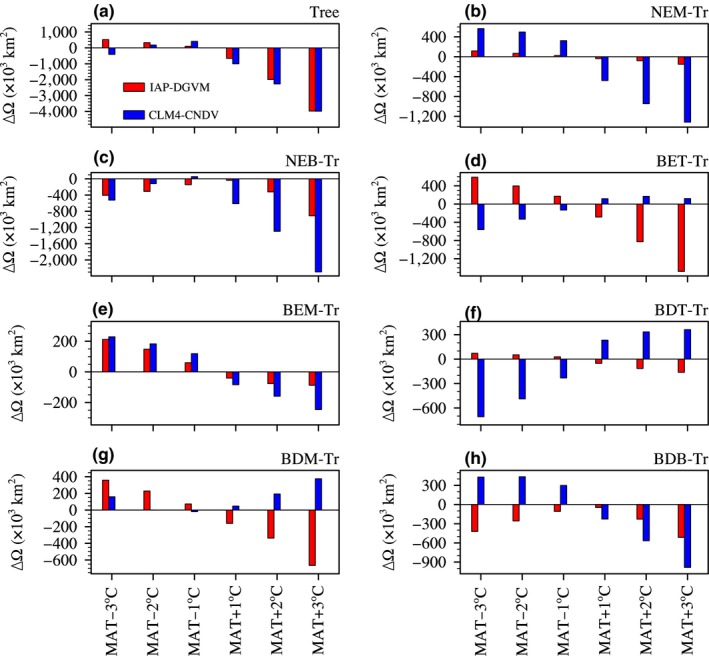
Comparison of the global area change (ΔΩ; ×10^3^ km^2^) for seven tree PFTs in six temperature change sensitivity tests

Temperature change may result in changes in forest ecosystem structures due to changes in GPP and population dynamics among different PFTs. In IAP‐DGVM and CLM4‐CNDV, there are seven tree PFTs, and different forest types are often expected to have various sensitivities to climate change. To explore how different tree PFTs respond to temperature change, the simulated changes in global area (ΔΩ; km^2^) for the seven tree PFTs are shown in Figure 3.

Overall, for two models, warming resulted in a reduction in the total area of trees around the globe, while cooling led to a slight increment in global area of trees, except in the case of MAT − 3°C for CLM4‐CNDV (Figure 3a). Furthermore, warming had a larger impact on the ΔΩ for trees than cooling. For boreal forests (NEB‐Tr and BDB‐Tr), warming consistently decreased their global areas (Ω; km^2^) in the two models (Figure 3c,h). When MAT declined, the Ωs of NEB‐Tr and BDB‐Tr from IAP‐DGVM decreased; however, in CLM4‐CNDV, cooling reduced NEB‐Tr's Ω (except in the case with MAT − 1°C) but increased BDB‐Tr's Ω. The combination of changes in the Ωs of NEB‐Tr and BDB‐Tr led to a reduction in boreal forest areas in both the two models.

In the two models, temperature increase had a negative impact on the global areas of NEM‐Tr and BEM‐Tr (Figure 3b,e). For the third temperate tree PFT, BDM‐Tr, the two models had opposite performance to warming (Figure 3g). Meanwhile, for BET‐Tr and BDT‐Tr, the dominant tree PFTs in tropical forests, the responses of their global areas to temperature changes were totally distinct between two models. In IAP‐DGVM, both BET‐Tr's Ω and BDT‐Tr's Ω had positive responses to decreasing temperature and negative relationships with warming, while in CLM4‐CNDV, the results were opposite (Figure 3d,f). It was probably because of differences in population dynamics schemes and photosynthesis parameterizations between models.

#### 4.1.2 Tree fractional coverage change and its influencing factors

Comparison of different temperature sensitivity tests showed some common points, e.g., the negative relationship between GPP_eco_ and warming, as well as the similar response to increasing or decreasing temperature for a given tree PFT. In the following, the cases with MAT ± 1°C were used to investigate the difference in *F*_tree_ (Δ*F*_tree_, %; *F*_tree_ in cases of temperature change—*F*_tree_ in the control simulation (*F*_tree,ctrl_) for each model) due to temperature change in different regions and to identify the influencing factors for the two models.

##### The global distribution of tree fractional coverage change

**Figure 4:**
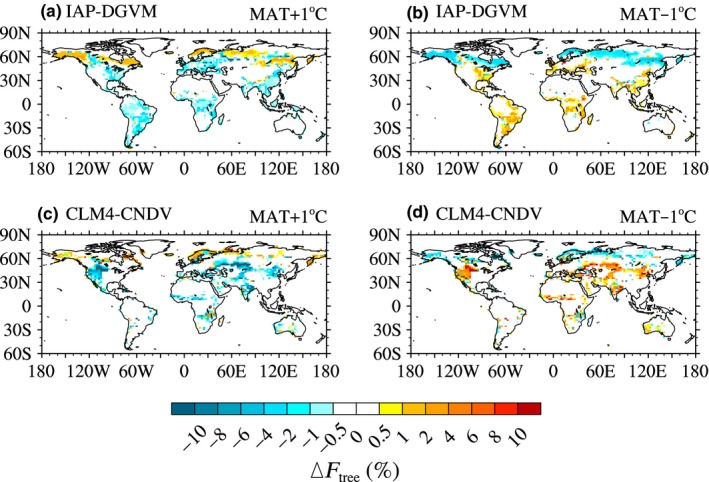
Global distribution of changes in tree fractional coverage (Δ*F*_tree_; %) due to temperature increasing or decreasing by 1°C for (a, b) IAP‐DGVM and (c, d) CLM4‐CNDV

Figure 4 shows the global distribution of Δ*F*_tree_ simulated by the two models, and only grid cells with |Δ*F*_tree_| > 5‰ are shown. Increasing temperature favored most boreal forests, especially in IAP‐DGVM (Figure 4a), but led to declines of *F*_tree_ in temperate and tropical regions. For IAP‐DGVM, over most regions south of 45°N, increasing temperature slightly decreased *F*_tree_ (−2% < Δ*F*_tree_ < 0), such as the Amazon and Central Africa rainforests, Indonesia, and southern China. Similar to IAP‐DGVM, *F*_tree_ in some boreal regions had a positive sensitivity to rising temperature in CLM4‐CNDV (but the sensitive areas were smaller; Figure 4c), and reduction in *F*_tree_ mainly occurred in arid or semiarid regions, such as the western United States and the marginal zone of Central Africa rainforests.

The case of cooling had similar sensitive areas to the case of warming, except some areas of Europe and the core areas of tropical rainforests (e.g., the center of the Amazon and Indonesian islands) in IAP‐DGVM (Figure 4a vs. b). Opposite to warming, temperate and tropical forests benefited from cooling in the IAP‐DGVM simulation, and the *F*_tree_ of boreal forests dropped by up to 4% (Figure 4b), in accordance with the conclusion in Figure 3. Similar to IAP‐DGVM, the result in the case of MAT − 1°C contradicted that in the MAT + 1°C experiment in CLM4‐CNDV (Figure 4c vs. d). Furthermore, in the cooling experiments, although the sensitive area in CLM4‐CNDV was smaller, the amplitude of Δ*F*_tree_ was larger, which may be due to the higher standard deviation of GPP in CLM4‐CNDV.

##### The factors influencing tree fractional coverage change

**Figure 5:**
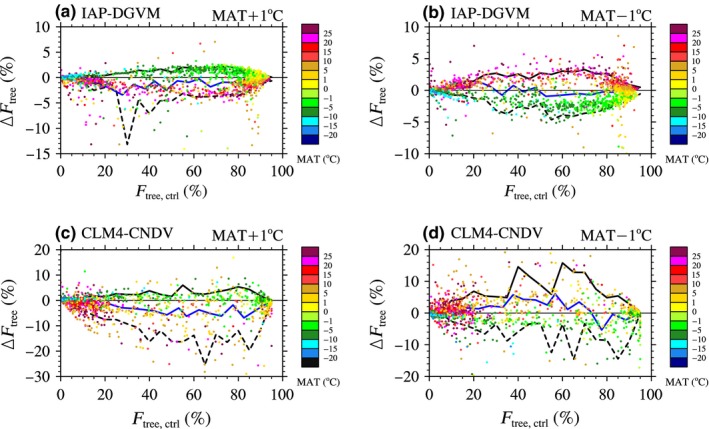
The dependence of the change in tree fractional coverage (Δ*F*_tree_; %) on the mean annual temperature (MAT; °C) and tree fractional coverage in the control simulation (*F*_tree,ctrl_; %) in the cases of MAT ± 1°C for (a, b) IAP‐DGVM and (c, d) CLM4‐CNDV. The blue line indicates the area‐averaged Δ*F*_tree_, and the black solid line and dashed line denote the 10th and 90th percentiles, respectively

As shown in Figure 4, forests in different regions might have different responses to temperature change, not only in the direction but also in the amplitude of the *F*_tree_ change. Are there any relationships between Δ*F*_tree_ and the local climate conditions or forest ecosystem characteristics? To answer this question, the relationship between Δ*F*_tree_ and MAT as well as *F*_tree,ctrl_ was investigated (Figure 5). Globally, warming led to negative area‐averaged Δ*F*_tree_ ($\bar{\Delta F_{\mathrm{tree}}}$; %) in any case of *F*_tree,ctrl_ for both models (the blue lines in Figure 5a,c), while the effects of decreasing temperature on $\bar{\Delta F_{\mathrm{tree}}}$were different between the two models (the blue lines in Figure 5b,d). When reducing MAT by 1°C, the area‐averaged *F*_tree_ ($\bar{F_{\mathrm{tree}}}$; %) increased in areas with *F*_tree,ctrl_ < 32% and decreased in regions with *F*_tree,ctrl_ > 50% in the IAP‐DGVM simulation (Figure 5b); however, for CLM4‐CNDV, $\bar{F_{\mathrm{tree}}}$increased when 0 < *F*_tree,ctrl_ < 72% ($\bar{\Delta F_{\mathrm{tree}}}$was approximately 7% when *F*_tree,ctrl_ was 55%), and then, with *F*_tree,ctrl_ > 72%, $\bar{F_{\mathrm{tree}}}$decreased due to decreasing temperature (Figure 5d).

In the IAP‐DGVM simulations, there were two distinct tendencies in the relationship between Δ*F*_tree_ and *F*_tree,ctrl_, and these tendencies depended on MAT (Figure 5a,b). When MAT increased by 1°C, *F*_tree_ increased (Δ*F*_tree_ > 0) in most grid cells with MAT < 0°C and decreased (Δ*F*_tree_ < 0) in most grid cells with MAT > 0°C (Figure 5a); however, Figure 5b shows that forests in warm regions benefited from cooling, in accordance with Figure 4. Figure 5a and b illustrates that the most impacted forest ecosystems were in regions with *F*_tree,ctrl_ ~ (60%, 80%; the absolute value of Δ*F*_tree_ (|Δ*F*_tree_|) was almost 4%). CLM4‐CNDV was similar to IAP‐DGVM, although the boundaries of |Δ*F*_tree_| between regions with MAT > 0 and MAT < 0 were not obvious, but |Δ*F*_tree_| was larger in CLM4‐CNDV (Figure 5c–d). Overall, MAT determined whether Δ*F*_tree_ was positive or negative, and the amplitude of Δ*F*_tree_ was relative to *F*_tree,ctrl_.

To quantitatively explain the dependence of Δ*F*_tree_ on MAT and *F*_tree,ctrl_, the correlation coefficient (*R* ^2^) was calculated (see Appendices S2 and S3). Because Figure 5 demonstrates that whether Δ*F*_tree_ was positive or negative largely depended on the MAT value, the simulation results were classified into two groups based on the MAT value (>0 or <0) for each case. Grid cells with an absolute value of MAT (|MAT|) less than 1°C in the control simulations were excluded from the analysis. Three regression equations were used to describe the separate and combined effects of MAT and/or *F*_tree,ctrl_ on Δ*F*_tree_. Normalization of MAT (MAT′; −1 ≤ MAT′ ≤ 1) was performed before regression, i.e., MAT′ = MAT/|MAT|_max_, where |MAT|_max_ was the maximum absolute value of MAT around the globe. Δ*F*_tree_ and *F*_tree,ctrl_ were used in decimal form rather than as percentages (%). There were similar phenomena in the IAP‐DGVM and CLM4‐CNDV simulation results, i.e., for the two models: (1) In all the cases, although Δ*F*_tree_ had a significant relationship with MAT′ (all cases had *p* < .0001 except one case with *p* < .01), it was more dependent on *F*_tree,ctrl_. For example, in the IAP‐DGVM simulations, when MAT decreased by 1°C, in areas with MAT <  0, MAT′ accounted for approximately 22.9% of the variation in Δ*F*_tree_, while *F*_tree,ctrl_ explained approximately 62.4% of the variation in Δ*F*_tree_, and the combined effects of MAT′ and *F*_tree,ctrl_ were approximately 64.2%; (2) when warming, grid cells with MAT ≥ 0 were more sensitive to *F*_tree,ctrl_ and MAT′ because regions with MAT ≥ 0 had larger *R* ^2^ than grid cells with MAT < 0 in regression Equation 1; on the other hand, when MAT decreased, forest ecosystems with MAT < 0 usually had strong responses to temperature and forest structure. Moreover, it was shown that forest ecosystems described by IAP‐DGVM were more dependent on *F*_tree,ctrl_ and MAT′ (*R* ^2^ from IAP‐DGVM was larger than that from CLM4‐CNDV for the same cases). For MAT ± 2°C or MAT ± 3°C, similar conclusions were obtained, so the results are not shown here.

**Figure 6:**
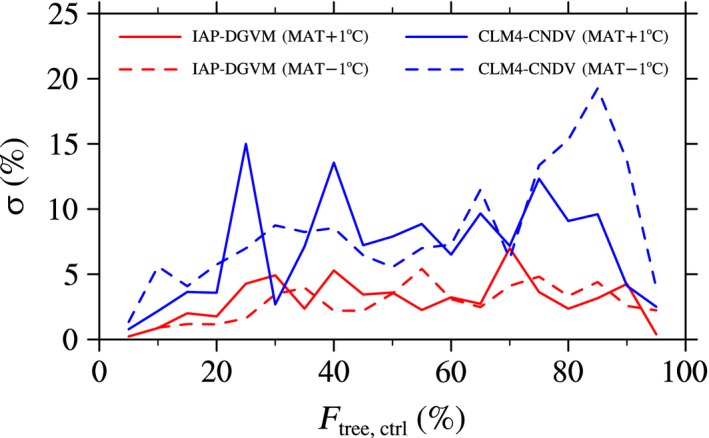
Dependence of the standard deviation of Δ*F*_tree_ (σ; %) on tree fractional coverage (*F*_tree,ctrl_; %) simulated by IAP‐DGVM and CLM4‐CNDV in the case of MAT ± 1°C

Compared with IAP‐DGVM, Δ*F*_tree_ in CLM4‐CNDV varied over a wide range, especially for forest ecosystems with *F*_tree,ctrl_ ~ (25%, 85%; Figure 5). To determine which types of forest ecosystems have large change in Δ*F*_tree_ for the two models, the relationship between *F*_tree,ctrl_ and global area‐averaged standard deviation of Δ*F*_tree_ (σ; %) was analyzed (Figure 6). The results showed that forest ecosystems simulated by CLM4‐CNDV usually had larger σ when the temperature varied. Except for the case with *F*_tree,ctrl_ at approximately 70%, σ from IAP‐DGVM was almost less than 5%, while in the CLM4‐CNDV cases, the maximum σ reached approximately 15% (*F*_tree,ctrl_ ~ 25%) and 20% (*F*_tree,ctrl_ ~ 85%) for warming and cooling, respectively (Figure 6). These differences may be due to the larger change in ΔGPP in CLM4‐CNDV.

### 4.2 The effects of precipitation change on forest ecosystems

**Figure 7:**
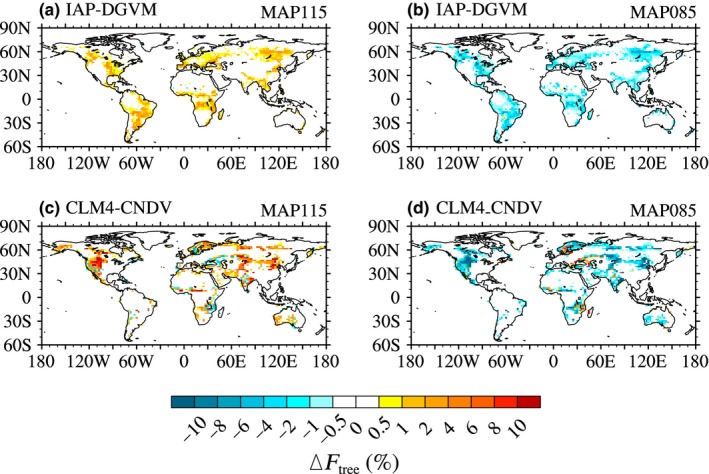
Global distribution of changes in tree fractional coverage (Δ*F*_tree_; %) due to precipitation increasing or decreasing by 15% for (a, b) IAP‐DGVM and (c, d) CLM4‐CNDV

Precipitation is another key factor that influences the vegetation distribution and ecosystem structure; therefore, its effects on Δ*F*_tree_ were investigated in the following. Figure 7 shows the global distribution of tree fractional coverage change due to precipitation change from IAP‐DGVM and CLM4‐CNDV, and following Figure 4, only grid cells with |Δ*F*_tree_| greater than 5‰ were shown. Compared with the cases of temperature change, the responses of forest ecosystems to mean annual precipitation (MAP) change were uniform, i.e., increased MAP led to globally increased *F*_tree_, while reduced MAP led to decreased *F*_tree_. However, the sensitive regions varied slightly between IAP‐DGVM and CLM4‐CNDV. In IAP‐DGVM, large changes in *F*_tree_ occurred in eastern North America, northern Asia, and most regions in South America (Figure 7a,b). However, in the CLM4‐CNDV simulations, the sensitive areas mainly covered western North America, Central Asia, and the peripheral areas of the core forests (e.g., the southeast of Central Africa; Figure 7c,d).

**Figure 8:**
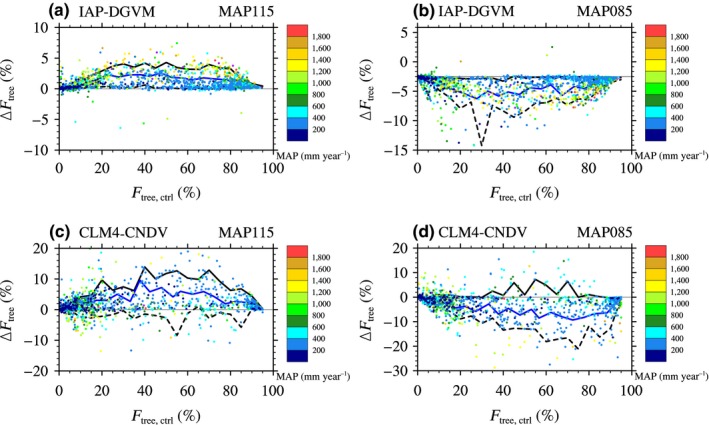
The dependence of the change in tree fractional coverage (Δ*F*_tree_; %) on the mean annual precipitation (MAP; mm/year) and tree fractional coverage in the control simulation (*F*_tree,ctrl_; %) in the cases of MAP increasing and decreasing by 15% for (a, b) IAP‐DGVM and (c, d) CLM4‐CNDV. The blue line indicates the area‐averaged Δ*F*_tree_. The black solid line and dashed line denote the 10th and 90th percentiles, respectively

In the responses of *F*_tree_ to MAP change, CLM4‐CNDV also had larger Δ*F*_tree_ than IAP‐DGVM (Figure 8). When increasing MAP by 15%, larger Δ*F*_tree_ occurred in areas with approximately 30% < *F*_tree_ < 80% in both models. However, in the case of decreasing MAP, obvious Δ*F*_tree_ appeared in the grid cells with approximately 20% < *F*_tree_ < 45% in IAP‐DGVM, while the sensitive regions were areas with approximately 60% < *F*_tree_ < 85% in CLM4‐CNDV.

**Figure 9:**
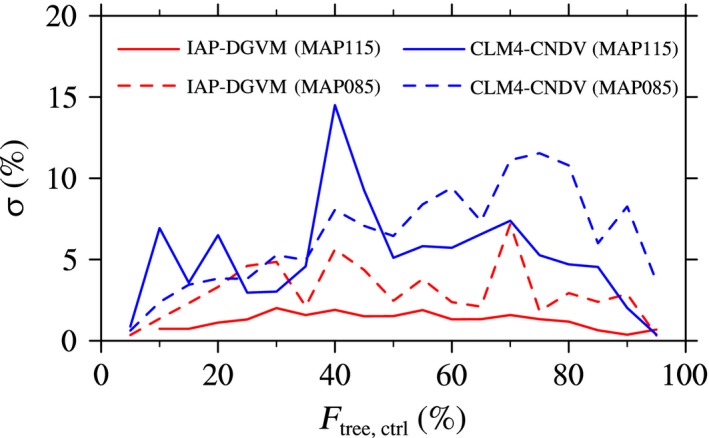
Dependence of the standard deviation of Δ*F*_tree_ (σ; %) on tree fractional coverage (*F*_tree,ctrl_; %) simulated by IAP‐DGVM and CLM4‐CNDV in the cases of MAP increasing or decreasing by 15%

Similarly, to further investigate the influences of MAP and *F*_tree,ctrl_ on Δ*F*_tree_, the correlation coefficients between Δ*F*_tree_ and *F*_tree,ctrl_, as well as MAP, were calculated (see Appendices S4 and S5). In the same way, normalization of MAP (mm/year; i.e., MAP′ = MAP/MAP_max_, where MAP_max_ was the maximum value of MAP around the globe) was performed before regression. Furthermore, Δ*F*_tree_ and *F*_tree,ctrl_ were used in decimal form rather than percentages (%).Δ*F*_tree_ had a significant relationship with *F*_tree,ctrl_ (*p* < .0001) and MAP′ (*p *< .0001), especially with *F*_tree,ctrl_, for both IAP‐DGVM and CLM4‐CNDV. Δ*F*_tree_ in the case of increasing MAP had greater dependence on *F*_tree,ctrl_ than cases with decreasing MAP (*R* ^2^ = .377 vs. .191 in IAP‐DGVM; *R* ^2^ = .181 vs. .154 in CLM4‐CNDV). Additionally, the Δ*F*_tree_ simulated by IAP‐DGVM had much stronger sensitivity to *F*_tree,ctrl_ and MAP′ than in the CLM4‐CNDV simulations. The relationship between the standard deviation of Δ*F*_tree_ and *F*_tree,ctrl_ was also considered. Similar to the cases of temperature change, σ in CLM4‐CNDV was generally larger than that in IAP‐DGVM for most forest ecosystems when MAP changed (Figure 9). For IAP‐DGVM, σ in the case of decreasing MAP was higher than σ in the case of increasing MAP for all groups of forest ecosystems, especially regions with *F*_tree,ctrl_ ~ 70%. However, for CLM4‐CNDV, grid cells with *F*_tree,ctrl_ < 48% had larger σ in the case of increasing MAP (except for areas with 25% < *F*_tree,ctrl_ < 36%), especially with *F*_tree,ctrl_ ~ 40%, whereas in the decreasing MAP sensitivity test, higher σ occurred in regions with 70% < *F*_tree,ctrl_ < 82%.

### 4.3 The effects of CO_2_ concentration change on forest ecosystems

**Figure 10:**
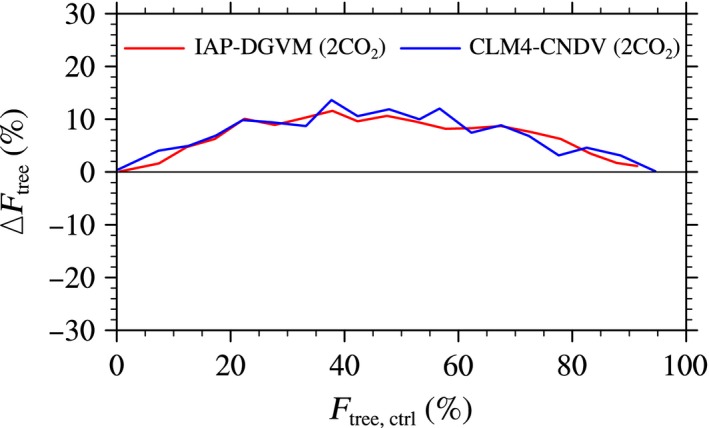
The relationship between tree fractional coverage (*F*_tree,ctrl_; %) and the change in tree fractional coverage (Δ*F*_tree_; %) in the cases of doubled concentration (2CO_2_) simulated by IAP‐DGVM and CLM4‐CNDV

Changes in the carbon dioxide level have attracted attention because increasing CO_2_ concentration not only results in global warming but also increases carbon fertilization. In this work, increasing CO_2_ does not lead to rising temperature, i.e., only carbon fertilization effects were considered. Figure 10 shows the relationship between *F*_tree,ctrl_ and area‐averaged Δ*F*_tree_ in the sensitivity tests with doubled concentration (2CO_2_) simulated by IAP‐DGVM and CLM4‐CNDV. It was shown that (1) the simulated *F*_tree_ in the two models had a positive response to CO_2_ concentration; (2) when doubling the CO_2_ concentration, ecosystems with 35% < *F*_tree,ctrl_ < 40% had the strongest sensitivity to CO_2_ change, Δ*F*_tree_ reached approximately 12% and 14% for IAP‐DGVM and CLM4‐CNDV, respectively.

## 5 Conclusions and Discussion

Forests are particularly vulnerable to changing environmental conditions due to the longevity of tree species (Kräuchi, 1993). However, climate change effects on forests may also be subtle, affecting individual tree growth and forest composition and structure from years to decades (Pederson et al., 2015).

In this study, the responses of forest ecosystems to changes in climate and CO_2_ concentration were investigated by IAP‐DGVM coupled with CLM3 and CLM4‐CNDV. In the temperature change sensitivity tests, it was shown that (1) the two models had different sensitive regions to temperature change, i.e., in IAP‐DGVM, the most sensitive areas were distributed in the core areas of forests, especially in boreal forests, while in CLM4‐CNDV, the most influenced regions were distributed in the transitional areas of boreal forests, the peripheral zones of tropical forests, and some semiarid or arid regions; (2) because warming led to stronger respiration and drier soil moisture, $\bar{{\text{GPP}}_{\mathrm{eco}}}$simulated by IAP‐DGVM and CLM4‐CNDV decreased with increasing MAT, which may be the main cause of the reduction in $\bar{F_{\mathrm{tree}}}$in warming cases; however, in the three cases with declining MAT, the trends of $\bar{\Delta {\text{GPP}}_{\mathrm{eco}}}$ were opposite between the two models, which partly accounted for the different responses of some tree PFTs to cooling (such as BET‐Tr, BDT‐Tr, and BDB‐Tr); (3) for MAT ± 1°C in both models, warming favored boreal forests, whereas cooling was beneficial to temperate and tropical forests; moreover, the difference in tree fractional coverage Δ*F*_tree_ and its global area‐averaged standard deviation from CLM4‐CNDV was larger than those in IAP‐DGVM; (4) Δ*F*_tree_ had a significant dependence on the local temperature and forest ecosystem structure: MAT largely determined whether Δ*F*_tree_ was positive or negative, while *F*_tree_ determined the amplitude of Δ*F*_tree_ around the globe, and such the dependence was stronger in IAP‐DGVM.

Compared with the temperature change, the responses of forests to precipitation and CO_2_ concentration changes were more uniform, i.e., *F*_tree_ increased with precipitation and CO_2_ concentration around the globe. The regions sensitive to increasing and decreasing MAP were different. Areas with 30% < *F*_tree_ < 60% (in IAP‐DGVM) or semiarid and arid regions (in CLM4‐CNDV) had strong sensitivity to increasing MAP; however, as MAP decreased, *F*_tree_ in areas with large *F*_tree_ decreased remarkably in IAP‐DGVM, while *F*_tree_ in semiarid and arid regions in CLM4‐CNDV dropped significantly. Similar to the temperature change simulations, Δ*F*_tree_ was more dependent on *F*_tree,ctrl_ than MAP, and the standard deviations of Δ*F*_tree_ in CLM4‐CNDV were higher than those from IAP‐DGVM. For the CO_2_ concentration simulations, both DGVMs captured the CO_2_ fertilization effects.

As shown in Figure 3, tropical PFTs had opposite responses to temperature change between two models. Our other research showed that such distinctions were likely to result from the differences in seedling establishment scheme and photosynthesis parameterization (see Appendice S1). IAP‐DGVM explicitly considers the impact of soil moisture on the establishment rates of woody PFTs. When temperature decreased, lower evapotranspiration increased soil moisture, not only benefiting seedling establishment rates which increased tree population densities, but also improving the maximum rate of carboxylation (*V*_max_) and GPP_eco_ (Figure 2), leading to individual growth. As a result, the fractional coverage of tropical forests increased. However, if not considering the soil moisture influences on establishment rates in IAP‐DGVM (Equation S6; like the establishment parameterization in CLM4‐CNDV), similar results with CLM4‐CNDV were found, i.e., the tropical tree population densities would decrease in the case of cooling. Therefore, introducing the effects of soil moisture on establishment rates directly accounted for the different vegetation responses to climate change. As to the fall in GPP simulated by CLM4‐CNVD in the case of cooling, it was mainly because of complicated nitrogen limitation. In CLM4‐CNDV, *V*_max_ also varies with foliage nitrogen concentration and specific leaf area (SLA, assumed to increase linearly with cumulative LAI). Such complicated nitrogen influences exceeded the positive effects from moister soil on *V*_max_ and led to GPP_eco_ decline when temperature decreased.

In IAP‐DGVM, the widest range of Δ*F*_tree_ appeared in the grid cells with 60% < *F*_tree,ctrl_ < 80%. However, in CLM4‐CNDV, Δ*F*_tree_ varied over a large range, as shown by the smaller number of grid cells with 25% < *F*_tree,ctrl_ < 85% (Figure 5). In addition to the differences in GPP variance, due to the significant dependence of Δ*F*_tree_ on *F*_tree,ctrl_, differences in the simulated *F*_tree,ctrl_ accounted for the discrepancies in Δ*F*_tree_ between the two models. The results showed that excluding grid cells with *F*_tree,ctrl_ < 5%, approximately 19.0% and 31.2% of the grid cells fell in the intervals *F*_tree,ctrl_ < 20% and *F*_tree,ctrl_ > 85% in IAP‐DGVM, whereas in CLM4‐CNDV, the percentages reached approximately 16.0% and 64.5%, respectively (Figure S2; to concentrate on the core areas of forests, only grid cells with *F*_tree,ctrl_ > 5% were considered, and in the two models, *F*_tree,ctrl_ is assumed not to exceed 95% in each grid cell; therefore, there were no results when *F*_tree,ctrl_ < 5% or *F*_tree,ctrl_ > 95%). The combination of the differences in the simulated *F*_tree,ctrl_ and the dependence of Δ*F*_tree_ on *F*_tree,ctrl_ largely accounted for the differences in Δ*F*_tree_ and its standard deviation in these two models.

**Figure 11:**
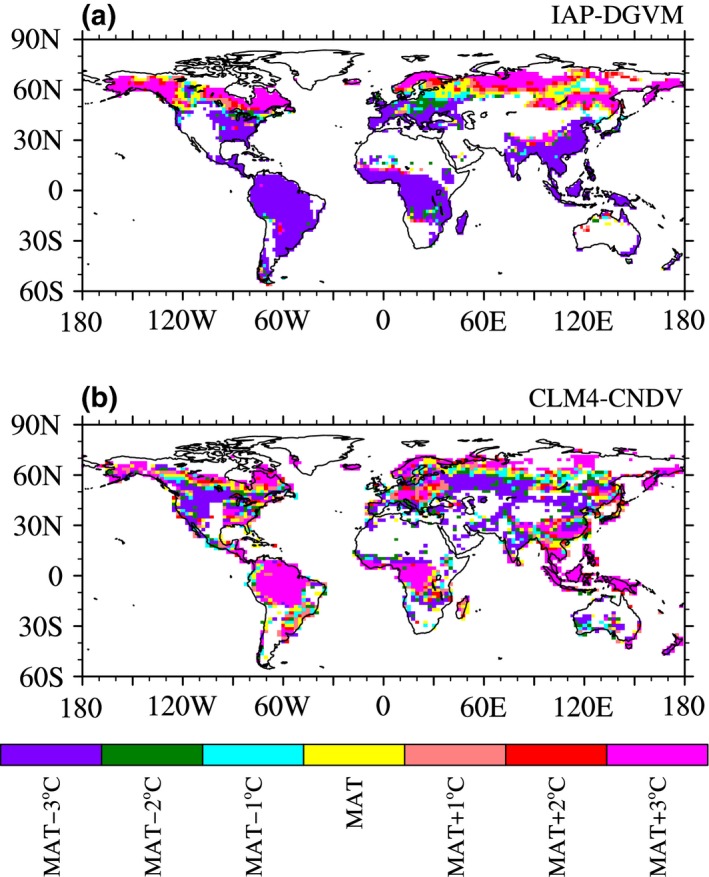
The global distribution of the optimal temperature change (relative to the current temperature) in (a) IAP‐DGVM and (b) CLM4‐CNDV

As discussed in previous research, the responses of forest ecosystems are spatially heterogeneous and partly uncertain (Mekonnen et al., 2016; Williams et al., 2010; Willis et al., 2012). To further investigate the differences in the response of forest ecosystems to temperature change in different regions, the optimal temperature change (relative to the current temperature) was defined as temperature condition in the seven temperature sensitivity tests under which *F*_tree_ was the largest. Only grid cells with *F*_tree,ctrl_ greater than 1% were considered. Large discrepancies existed in the global distribution of the optimal temperature between IAP‐DGVM and CLM4‐CNDV (Figure 11). In IAP‐DGVM, most boreal forests had their largest *F*_tree_ at MAT + 3°C, while for some boreal regions, the optimal temperature conditions were MAT − 1°C, MAT, or MAT + 2°C. In accordance with Figure 4a, temperate and tropical forests benefited from decreased temperature in IAP‐DGVM, and *F*_tree_ reached the maximum value when MAT decreased by 3°C. For CLM4‐CNDV, the advantage of warming appeared in a smaller range of boreal forests, consistent with Figure 4b, and the maximum *F*_tree_ was reached at MAT − 2°C, MAT − 1°C or MAT in many boreal grid cells. In the arid and semiarid regions (e.g., the western USA) and transitional zones of forests (e.g., the peripheral areas of the tropical rainforests in Central Africa), decreased temperature was good for forest coverage, and *F*_tree_ was largest at MAT − 3°C because cooling relieved drought or reduced respiration, decreasing tree mortality in these regions, which was somewhat in accordance with Williams et al. (2010). The tropical forests in CLM4‐CNDV mostly had the largest *F*_tree_ in the case of MAT + 3°C; however, due to *F*_tree,ctrl_ being close to the upper 95% limit provided in the models, the increment of *F*_tree_ was small in these areas.

This work provided valuable ideas to investigate the responses of forest ecosystems to climate change and several vital clues to explore the uncertainties in the current vegetation dynamic models. In the following work, the combined effects of changes in temperature and precipitation on vegetation will be considered.

## Conflict of Interest

None declared.
