# Supplementary material for: Evaluating the responses of forest ecosystems to climate change and CO2 using dynamic global vegetation models
Source: Ecol Evol. 2017 Jan 17;7(3):997–1008. doi: 10.1002/ece3.2735 (PMC5288257; doi:10.1002/ece3.2735)
Supplement: Supplementary file 1 [file ECE3-7-997-s001.doc]

**Appendix A. The definition of fractional coverage and its dependent processes**

As mean field models, both IAP-DGVM1.0 and CLM4-CNDV ignore the differences among individuals within the same woody PFT, and define fractional coverage (*F*; %) as

*F* = *σ*×*n* (A1)

where *σ* (m^2^ per individual) and *n* (individuals m^-2^) denote the averaged individual crown area and the population density for a given woody PFT, respectively.

1. **The calculation of individual crown area**


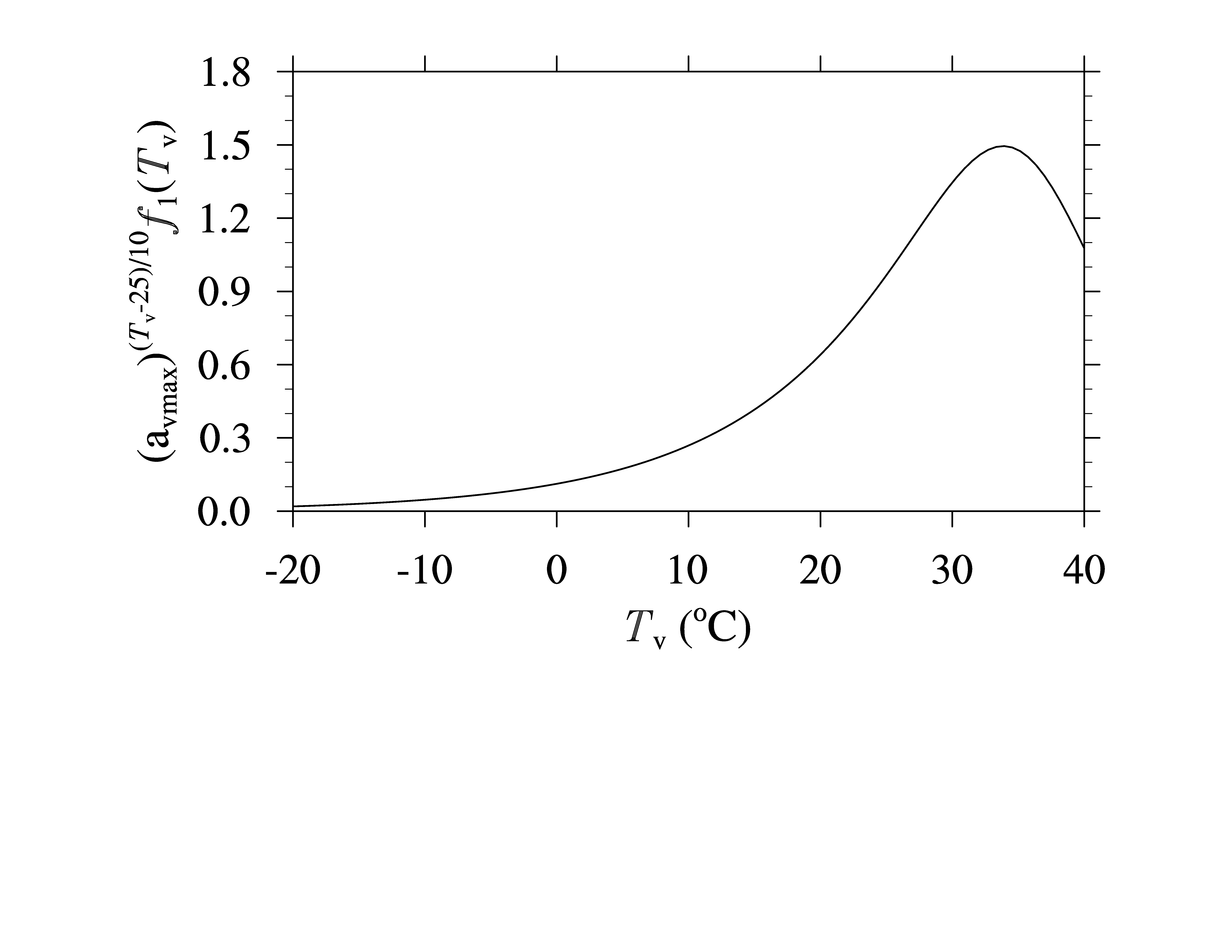


**Fig. A1.** The dependence of the maximum rate of carboxylation on vegetation temperature

Individual crown area depends on NPP amount and NPP allocation among individual leaf, stem and root. NPP amount is determined by photosynthesis and respiration which are two processes having close relationship with climate and CO_2_. In this work, leaf photosynthesis in IAP-DGVM is determined by the internal leaf CO_2_ concentration, atmospheric pressure, temperature, soil water, nitrogen limitation, as well as plant traits (see Eq. (8.2)-(8.10) in Oleson *et al.*, 2004 and section 2 in Zeng, 2008). For instance, the maximum rate of carboxylation (*V*_max_) is written as

$V_{\text{max}}=V_{\text{max25}}\text{(}a_{\text{vmax}}\text{)}^{\frac{T_{\text{v}}-25}{10}}f_{1}\text{(}T_{\text{v}}\text{)}g\text{(}\beta_{\text{t}}\text{)}f_{2}\text{(}N\text{)}$ (A2)

where *V*_max25_ is the prescribed value of *V*_max_ at 25°C, *a*_vmax_ is the *Q*_10_ parameter (equals to 2.4), *f*_1_(*T*_v_) and *g*(*β*_t_) denote the functions relative to leaf temperature (*T*_v_) and soil moisture (*β*_t_), respectively; *f*_2_(*N*) is the nitrogen limitation factor (a PFT-dependent constant, see Table 2 in Zeng, 2008). The dependence of *V*_max_ on *T*_v_ was shown in Fig. A1.

CLM4-CNDV uses the same ways to describe the impacts of leaf temperature and soil moisture on *V*_max_, however, it relates *V*_max25_ with changeable nitrogen concentration and introduces the role of day length. Therefore, *V*_max_ can be written as

$V_{\text{max}}=V_{\text{max25}}\text{(}a_{\text{vmax}}\text{)}^{\frac{T_{\text{v}}-25}{10}}f\text{(}T_{\text{v}}\text{)}g\text{(}\beta_{\text{t}}\text{)}h\text{(}DYL\text{)}$ (A3)

$V_{\text{max25}}=N_{a}M$ (A4)

$N_{a}=\frac{1}{CN_{L}SLA}$ (A5)

where *h*(*DYL*) is a function that scales *V*_max25_ for daylength and introduces seasonal variation to *V*_max_; *N*_a_ is the area-based leaf nitrogen concentration (gN m^-2^ leaf area), and inversely proportional to the specific leaf area (SLA; m^2^ leaf area g^-1^C); *M* and *CN*_L_ are relevant parameters (see section 8 in Oleson *et al*., 2010).

On the other hand, auto respiration usually includes maintenance respiration and growth respiration. In models, maintenance respiration is defined as a function of temperature and tissue nitrogen concentration for live biomass, while growth respiration is calculated as a constant factor times the carbon in new growth. In IAP-DGVM simulations, maintenance respiration rates for leaf and sapwood are based on vegetation temperature, and root respiration is relative to the weighted average of soil temperature in the top 25 cm of soil (Zeng *et al.*, 2014). However, in CLM4-CNDV, maintenance respiration rates for aboveground pools depend on the 2 meter air temperature, while rates for fine and coarse roots rely on soil temperature (Oleson *et al.*, 2010).

After NPP amount calculation, NPP allocation works and individual crown area is determined based on some empirical equations (see Eq. (11) in Zeng *et al.*, 2014 and Eq. (14.1) in Oleson *et al.*, 2010).

1. **The calculation of population density**

Population density (*n*) is another vital variable determining vegetation fractional coverage. In the two models, *n* is a prognostic variable, and depends on the establishment rate, the population decreases due to light competition, mortality and fire. The major difference between the two models is the establishment parameterization, while light competition, mortality and fire are almost the same (Oleson *et al.*, 2010; Zeng *et al.*, 2014).

In IAP-DGVM, establishment of woody vegetation refers to flowering, fertilization, seed production, germination, and the growth of tree seedlings. IAP-DGVM applies a hierarchy of tree-grass-shrub for the competition of establishment. Similar to LPJ model, a woody PFT (trees and shrubs) can establish in a grid cell if the required climate conditions are satisfied. If a tree PFT can be established, its establishment rate per year (Δ*n*_est,i_) is calculated as

$\text{Δ}n_{\text{est, }i}=\text{Δ}n_{\text{est, tree}}\frac{g_{i}}{\sum_{k=1}^{d_{\text{est, tree}}} g_{k}}S$, (A6)

$\text{Δ}n_{\text{est, tree}}=\text{Δ}n_{\text{estmax, tree}}(1-F_{\text{woody}})[1-\exp\text{(}-5(1-F_{\text{woody}})\text{)}]$, (A7)

$g_{i}=g_{i0}[\varepsilon_{0}+(1-\varepsilon_{0})F_{i}^{\alpha}]$, (A8)

where Δ*n*_est, tree_ is the total establishment rate of tree PFTs in the grid cell, depending on the maximum establishment rate of trees, Δ*n*_estmax, tree_ (0.24 individuals per m^2^), the space that is not occupied by the existing woody PFTs, (1−*F*_woody_), and the shading effects, 1−exp(−5(1−*F*_woody_)) (Sitch et al., 2003; Levis et al., 2004); *d*_est, tree_ is the number of established trees in the corresponding grid cell; *g_i_* denotes the capability of the *i*th woody PFT in establishment competition, related to the relative establishment potential, *g_i_*_0_ (PFT-dependent constant), and the vegetation states, e.g., in proportion to the fractional coverage of the *i*th woody PFT in the current year *F_i_* with the cluster effect factor (*α*), and the background establishment (*ε*_0_), which represents uncertainties such as the establishment from seeds produced in previous years or propagated from nearby grid cells; *S* describes the effects of soil moisture on establishment rate (see Eq. (5)-(7) in Song and Zeng, 2016).

In CLM4-CNDV, the population density increment due to establishment is calculated as Eq. (53) in Levis *et al.* (2004), which can also be written as Eq. (A6)-(A8) but with *g*_i0_ ≡ *ε* ≡ *S* ≡ 1. That is to say, CLM4-CNDV does not consider the impacts from soil water and current vegetation status (fractional coverage) on establishment rate, as well as the differences in establishment capability among different PFTs.

**Appendix B.** Regression equations between the change in tree fractional coverage and

tree fractional coverage as well as the mean annual temperature for IAP-DGVM

| **MAT+1°C** | | | | | | | |
| --- | --- | --- | --- | --- | --- | --- | --- |
| **Equation** | **b(1)** | **b(2)** | **b(3)** | **b(4)** | **b(5)** | **P** | **R^2^** |
| **MAT >= 0** |  |  |  |  |  |  |  |
| **Eq. 1** | 0.109*** | 0.010* | -0.105*** | -0.013*** | 0.004*** | <0.0001 | 0.339 |
| **Eq. 2** | 0.109*** | - | -0.105*** | - | 0.004 | <0.0001 | 0.332 |
| **Eq. 3** | - | 0.013** | - | -0.014** | -0.003** | <0.01 | 0.005 |
| **MAT < 0** |  |  |  |  |  |  |  |
| **Eq. 1** | 0.003 | -0.027ǂ | 0.013* | -0.025* | -0.005** | <0.0001 | 0.138 |
| **Eq. 2** | -0.0005 | - | 0.012* | - | -0.0002 | <0.0001 | 0.129 |
| **Eq. 3** | - | 0.009 | - | 0.019* | 0.007** | <0.0001 | 0.043 |
| **MAT-1°C** | | | | | | | |
| **Model** | **b(1)** | **b(2)** | **b(3)** | **b(4)** | **b(5)** | **P** | **R^2^** |
| **MAT >= 0** |  |  |  |  |  |  |  |
| **Eq. 1** | -0.083*** | -0.027*** | 0.079*** | 0.036*** | -0.011*** | <0.0001 | 0.271 |
| **Eq. 2** | -0.083*** | - | 0.079*** | - | -0.001* | <0.0001 | 0.201 |
| **Eq. 3** | - | -0.029*** | - | 0.037*** | -0.006*** | 0 | 0.059 |
| **MAT < 0** |  |  |  |  |  |  |  |
| **Eq. 1** | 0.062*** | 0.049*** | -0.079*** | 0.043*** | 0.008*** | <0.0001 | 0.642 |
| **Eq. 2** | 0.068*** | - | -0.079*** | - | -0.0007 | <0.0001 | 0.624 |
| **Eq. 3** | - | 0.018 | - | -0.022** | -0.018*** | <0.0001 | 0.229 |

**Note:** Eq. 1: ΔF_tree_ = b(1)×F_tree_^2^ + b(2)×MAT^’2^ + b(3)×F_tree_ + b(4)×MAT’ + b(5); Eq. 2: ΔF_tree_ = b(1)×F_tree_^2^ + b(3)×F_tree_ + b(5); Eq. 3: ΔF_tree_ = b(2)×MAT^’2^ + b(4)×MAT’ + b(5), where ΔF_tree_ is the change in tree fractional coverage in decimal form, F_tree_ is the tree fractional coverage in decimal form, and MAT’ is the normalized mean annual temperature. The coefficients of the regression equations are expressed as b. *** p < 0.0001，** p < 0.001，* p < 0.01, and ǂ p < 0.1 for the 95% confidence F-test.

**Appendix C.** Regression equations between the change in tree fractional coverage and

tree fractional coverage as well as the mean annual temperature for CLM4-CNDV

| **MAT+1°C** | | | | | | | |
| --- | --- | --- | --- | --- | --- | --- | --- |
| **Equation** | **b(1)** | **b(2)** | **b(3)** | **b(4)** | **b(5)** | **F_pval** | **R^2^** |
| **MAT >= 0** |  |  |  |  |  |  |  |
| **Eq. 1** | 0.251*** | -0.036 | -0.243*** | 0.019 | -0.001 | <0.0001 | 0.238 |
| **Eq. 2** | 0.253*** | - | -0.245*** | - | 0.001 | <0.0001 | 0.237 |
| **Eq. 3** | - | -0.140** | - | 0.109*** | -0.026*** | 0 | 0.030 |
| **MAT < 0** |  |  |  |  |  |  |  |
| **Eq. 1** | -0.060*** | -0.003 | 0.055*** | -0.002 | 0.0002 | <0.0001 | 0.058 |
| **Eq. 2** | -0.060*** | - | 0.055*** | - | 0.0003 | <0.0001 | 0.058 |
| **Eq. 3** | - | 0.006ǂ | - | 0.008* | 0.003*** | <0.0001 | 0.008 |
| **MAT-1°C** | | | | | | | |
| **Equation** | **b(1)** | **b(2)** | **b(3)** | **b(4)** | **b(5)** | **F_pval** | **R^2^** |
| **MAT >= 0** |  |  |  |  |  |  |  |
| **Eq. 1** | -0.182*** | -0.016 | 0.173*** | 0.009 | -0.002 | <0.0001 | 0.166 |
| **Eq. 2** | -0.180*** | - | 0.172*** | - | -0.0003 | 0 | 0.166 |
| **Eq. 3** | - | 0.050 | - | -0.049* | 0.015*** | <0.0001 | 0.015 |
| **MAT < 0** |  |  |  |  |  |  |  |
| **Eq. 1** | 0.181*** | 0.013* | -0.178*** | 0.014* | 0.002ǂ | 0 | 0.265 |
| **Eq. 2** | 0.177*** | - | -0.173*** | - | -0.0002 | <0.0001 | 0.263 |
| **Eq. 3** | - | -0.041*** | - | -0.050*** | -0.014*** | 0 | 0.066 |

**Note:** Eq. 1: ΔF_tree_ = b(1)×F_tree_^2^ + b(2)×MAT^’2^ + b(3)×F_tree_ + b(4)×MAT’ + b(5); Eq. 2: ΔF_tree_ = b(1)×F_tree_^2^ + b(3)×F_tree_ + b(5); Eq. 3: ΔF_tree_ = b(2)×MAT^’2^ + b(4)×MAT’ + b(5), where ΔF_tree_ is the change in tree fractional coverage in decimal form, F_tree_ is the tree fractional coverage in decimal form, and MAT’ is the normalized mean annual temperature. The coefficients of regression equations are expressed as b. *** p < 0.0001，** p < 0.001，* p < 0.01, and ǂ p < 0.1 for the 95% confidence F-test.

**Appendix D.** Regression equations between the change in tree fractional coverage and

tree fractional coverage as well as the mean annual precipitation for IAP-DGVM

| **MAP115** | | | | | | | |
| --- | --- | --- | --- | --- | --- | --- | --- |
| **Equation** | **b(1)** | **b(2)** | **b(3)** | **b(4)** | **b(5)** | **F_pval** | **R^2^** |
| **Eq. 1** | -0.085*** | -0.038*** | 0.076*** | 0.037*** | -0.002*** | 0 | 0.437 |
| **Eq. 2** | -0.085*** | - | 0.080*** | - | 0.0003 | 0 | 0.377 |
| **Eq. 3** | - | -0.073*** | - | 0.058*** | -0.0002 | <0.0001 | 0.140 |
| **MAP085** | | | | | | | |
| **Equation** | **b(1)** | **b(2)** | **b(3)** | **b(4)** | **b(5)** | **F_pval** | **R^2^** |
| **Eq. 1** | 0.144*** | 0.042*** | -0.130*** | -0.039*** | 0.002*** | <0.0001 | 0.202 |
| **Eq. 2** | 0.145*** | - | -0.135*** | - | -0.001 | 0 | 0.191 |
| **Eq. 3** | - | 0.103*** | - | -0.076*** | -0.002* | 0 | 0.040 |

**Note:** Eq. 1: ΔF_tree_ = b(1)×F_tree_^2^ + b(2)×MAP^’2^ + b(3)×F_tree_ + b(4)×MAP’ + b(5); Eq. 2: ΔF_tree_ = b(1)×F_tree_^2^ + b(3)×F_tree_ + b(5); Eq. 3: ΔF_tree_ = b(2)×MAP^’2^ + b(4)×MAP’ + b(5), where ΔF_tree_ is the change in tree fractional coverage in decimal form, F_tree_ is the tree fractional coverage in decimal form, and MAP’ is the normalized mean annual precipitation. The coefficients of regression equations are expressed as b. *** p < 0.0001，** p < 0.001，* p < 0.01, and ǂ p < 0.1 for the 95% confidence F-test.

**Appendix E.** Regression equations between the change in tree fractional coverage and

tree fractional coverage as well as the mean annual precipitation for CLM4-CNDV

| **MAP115** | | | | | | | |
| --- | --- | --- | --- | --- | --- | --- | --- |
| **Equation** | **b(1)** | **b(2)** | **b(3)** | **b(4)** | **b(5)** | **F_pval** | **R^2^** |
| **Eq. 1** | -0.199*** | 0.010 | 0.191*** | -0.008 | 0 | 0 | 0.181 |
| **Eq. 2** | -0.199*** | - | 0.189*** | - | 0 | <0.0001 | 0.181 |
| **Eq. 3** | - | -0.088*** | - | 0.042*** | 0.003*** | <0.0001 | 0.008 |
| **MAP085** | | | | | | | |
| **Equation** | **b(1)** | **b(2)** | **b(3)** | **b(4)** | **b(5)** | **F_pval** | **R^2^** |
| **Eq. 1** | 0.226*** | 0.021 | -0.225*** | 0.003 | 0 | <0.0001 | 0.155 |
| **Eq. 2** | 0.230*** | - | -0.226*** | - | 0 | 0 | 0.154 |
| **Eq. 3** | - | 0.161*** | - | -0.082*** | -0.003*** | 0 | 0.016 |

**Note:** Eq. 1: ΔF_tree_ = b(1)×F_tree_^2^ + b(2)×MAP^’2^ + b(3)×F_tree_ + b(4)×MAP’ + b(5); Eq. 2: ΔF_tree_ = b(1)×F_tree_^2^ + b(3)×F_tree_ + b(5); Eq. 3: ΔF_tree_ = b(2)×MAP^’2^ + b(4)×MAP’ + b(5), where ΔF_tree_ is the change in tree fractional coverage in decimal form, F_tree_ is the tree fractional coverage in decimal form, and MAP’ is the normalized mean annual precipitation. The coefficients of the regression equations are expressed as b. *** p < 0.0001，** p < 0.001，* p < 0.01, and ǂ p < 0.1 for the 95% confidence F-test.

**Appendix F.**


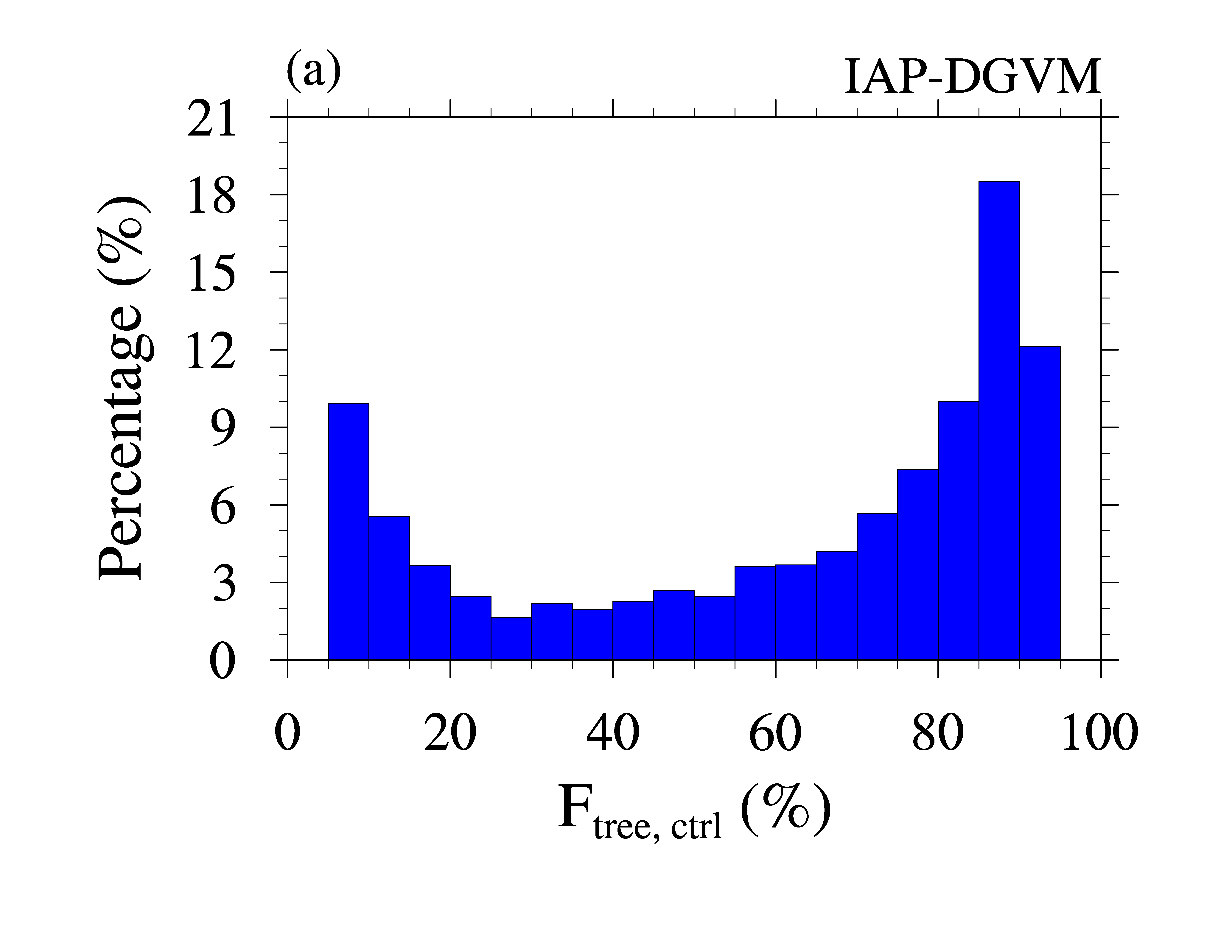


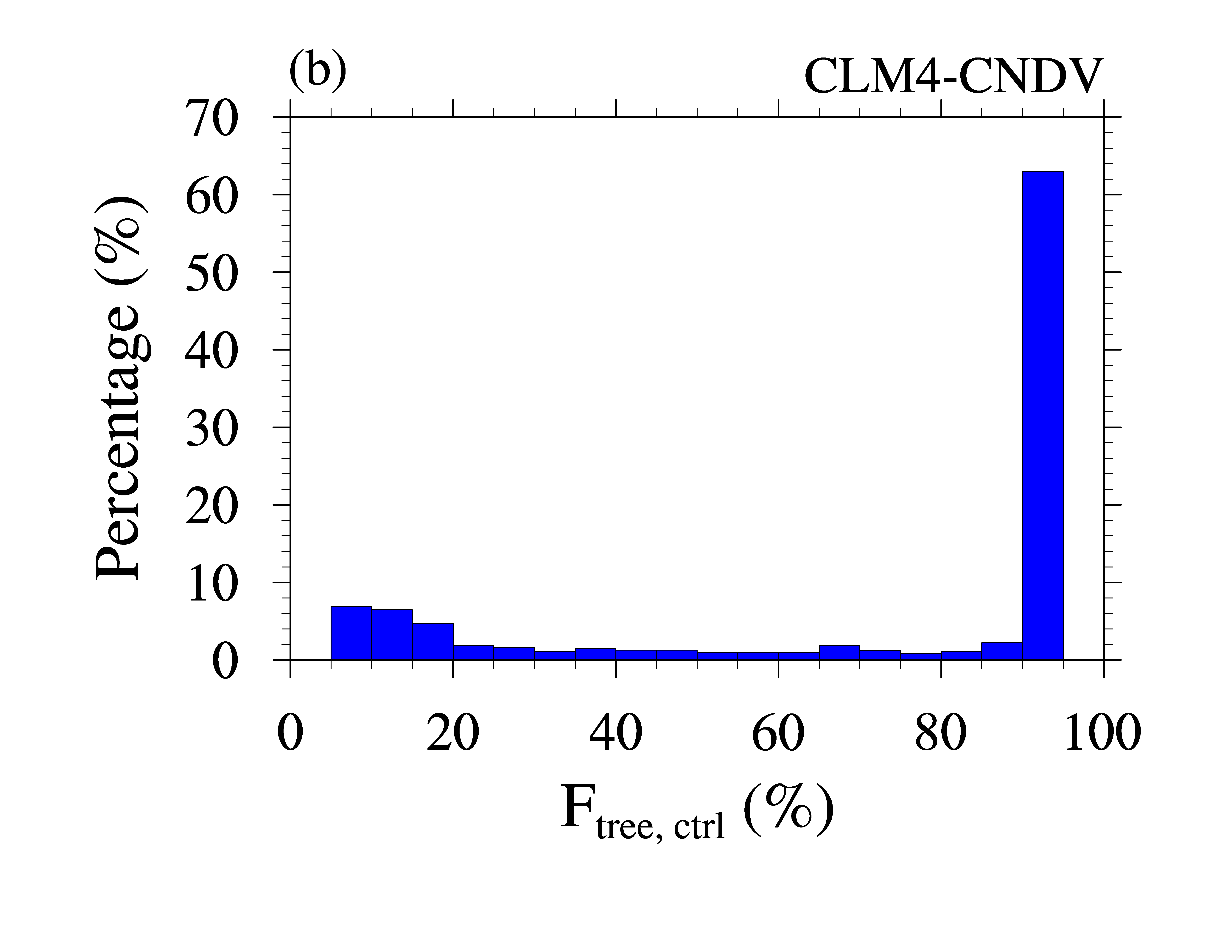


**Fig. A2.** The global spectrum distribution of tree fractional coverage in the control case (F_tree, ctrl_; %) for (a) IAP-DGVM and (b) CLM4-CNDV.
